# The Diagnostic Impact of Epigenomics in Pituicyte-derived Tumors: Report of an Unusual Sellar Lesion with Extensive Hemorrhage and Necrotic Debris

**DOI:** 10.1007/s12022-022-09727-z

**Published:** 2022-08-03

**Authors:** Matthias Dottermusch, Roman Rotermund, Franz L. Ricklefs, Annika K. Wefers, Wolfgang Saeger, Jörg Flitsch, Markus Glatzel, Jakob Matschke

**Affiliations:** 1 Institute of Neuropathology, University Medical Center Hamburg-Eppendorf, Martinistr. 52, 20246 Hamburg, Germany; 2 Center for Molecular Neurobiology (ZMNH), University Medical Center Hamburg-Eppendorf, Hamburg, Germany; 3 Department of Neurosurgery, University Medical Center Hamburg-Eppendorf, Hamburg, Germany; 4 Institute of Pathology, University Medical Center Hamburg-Eppendorf, Hamburg, Germany

**Keywords:** Posterior pituitary tumor, Pituicyte-derived tumor, Pituicyte tumor family, Oncocytic pituicytoma, Spindle cell oncocytoma, DNA methylation, Hemorrhage, Copy number alterations

The pituicyte tumor family, as defined in the 2022 WHO classification of pituitary tumors, includes pituicytoma (PITUI), granular cell tumor (GCT)/granular cell pituicytoma, spindle cell oncocytoma (SCO)/oncocytic pituicytoma and ependymal pituicytoma as distinct low-grade neoplasms of the neurohypophysis [1, 2]. Although considered benign, these tumors may show recurrences in up to two-thirds of cases [3]. Members of the pituicyte tumor family are recognized as pituicyte-derived neoplasms, demonstrated by their common expression of thyroid transcription factor 1 (TTF1) [3, 4]. Immunohistochemical, ultrastructural and molecular analyses of previous studies have suggested that PITUI, GCT and SCO represent subtypes of a single nosological entity [4, 5].

We here describe the role of epigenomic analyses in the diagnostic workup of a challenging sellar lesion identified in a 57-year-old male. The patient presented with generalized weakness and soreness, as well as lack of appetite and weight loss in the last 3 preceding months. Neurological symptoms comprised memory problems and difficulties focusing. Moreover, the patient noticed a progressive impairment in the left temporal visual field. Magnetic resonance imaging indicated a well circumscribed sellar mass with a maximum diameter of 2.2 cm (Fig. 1a). Basal blood serum levels of pituitary hormones (FSH, LH, ACTH, GH, TSH and PRL) were either greatly decreased or below detection levels at the time point of hospital admission, indicating a complete insufficiency of anterior pituitary hormone secretion. The patient was subjected to trans-sphenoidal surgery. Gross total resection of the sellar mass was achieved.

**Fig. 1:**
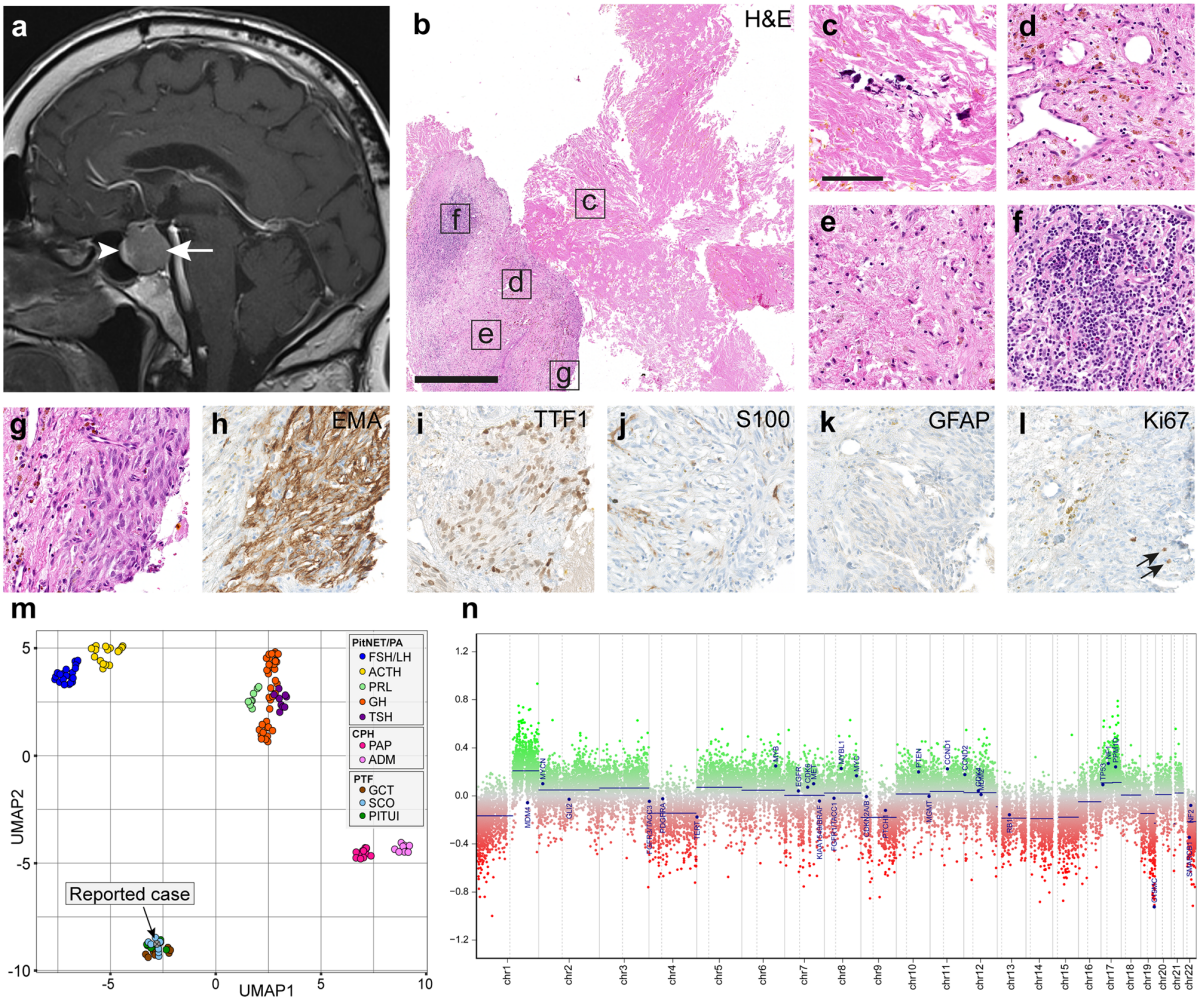
Radiological, histological, immunohistochemical and epigenomic findings. **a** Sagittal cranial MRI (*T*_1_-weighted image plus contrast) showing a sellar lesion of 2.2 × 2.1 × 2.1 cm in the posterior pituitary gland (white arrow). The lesion is well-circumscribed und shows faint homogeneous contrast enhancement, compared to the strong contrast enhancement of the anterior pituitary (white arrowhead). **b** Representative histological overview of the surgery specimen (H&E). Scale bar is 1 mm. **c**–**f** Non-neoplastic elements comprised necrotic debris and calcifications (**c**), golden-brown hemosiderin deposits in macrophages (**d**) as well as the extracellular space of a loose fibrillary matrix with low cellularity (**e**) and scattered lymphoid aggregates (**f**). **g**–**l** Eosinophilic spindle and epithelioid cells (**g**) with expression of EMA (**h**) and nuclear TTF1 (**i**) were found. S100 expression was partially visible (**j**). Staining for GFAP was negative (**k**). Less than 3% of nuclei stained positive for Ki67 (black arrows, (**l**)). Scale bar in c–l is 100 µm. **m** UMAP cluster analysis of methylation data including reference tumors of the posterior pituitary lobe (GSE185041, [4]) and further sellar neoplasms (GSE109381, [6]) demonstrated affiliation of the sample with the pituicyte tumor family. PitNET/PA: Pituitary neuroendocrine tumor/pituitary adenoma; FSH/LH: gonadotroph; ACTH: corticotroph; PRL: lactotroph; GH: somatotroph; TSH: thyrotroph; CPH: craniopharyngioma; PAP: papillary; ADM: adamantinomatous; PTF: pituicyte tumor family; GCT: granular cell tumor; SCO: spindle cell oncocytoma; PITUI: pituicytoma. **n** The copy number profile generated from global DNA methylation data showed diverse gains and losses of whole chromosomes and entire chromosome arms

Upon histological examination, we saw vast areas of necrotic debris, calcifications, abundant golden-brown hemosiderin deposits and a loose fibrillary matrix with low cellularity and scattered lymphoid aggregates (Fig. 1b–f). Moreover, we occasionally found small groups of polymorphic eosinophilic spindle and epithelioid cells, predominantly in fascicular formations (Fig. 1g). Some of these cells displayed clear cytoplasm, enlarged nuclei and sporadically visible nucleoli. Immunohistochemistry showed that these spindle cells prominently expressed EMA (Fig. 1h) and nuclear TTF1 (Fig. 1i). We saw partial immunopositivity for S100 in the spindle cells (Fig. 1j). Staining for GFAP was negative (Fig. 1k). The Ki67-immunolabeling demonstrated a low proliferative activity (< 3%, Fig. 1l). No mitotic figures were identified. The histomorphology and immunohistochemistry were suggestive of a pituicyte-derived origin of the spindle cells. However, due to the sparse presence of these cells and the overwhelming non-neoplastic changes, we concluded that histology did not allow the diagnosis of a neoplasm with certainty.

DNA was extracted from the sparsely present spindle cells, using carefully targeted punch biopsies of the formalin-fixed, paraffin-embedded tissue. The entire amount of extracted DNA (87.4 ng) was subjected to global DNA methylation profiling using the Illumina EPIC BeadChip array. The brain tumor methylation classifier (v11b4 and v12.5) [6] matched the sample to the methylation class “pituicytoma/granular cell tumor/spindle cell oncocytoma” (scores 0.95 and 0.99, respectively). UMAP cluster analysis confirmed epigenomic similarity with members of the pituicyte tumor family (Fig. 1m). A copy number profile exhibited diverse gains and losses of entire chromosomes or chromosomal arms (Fig. 1n), suggestive of neoplastic tissue.

In summary and combining all diagnostic layers, the case was classified as a low-grade neoplasm of the posterior pituitary lobe, which - based on histological features - was most compatible with a spindle cell oncocytoma/oncocytic pituicytoma, as per the 2022 WHO classification [1].

After surgery, the patient made a good recovery including visual improvement.

When approaching epigenomic analyses in unusual lesions of the posterior pituitary, careful interpretation of the results is advised. It has been demonstrated before that confident epigenomic distinction of normal posterior pituitary tissue and neoplasms thereof is currently not warranted [4]. Consequently, the brain tumor methylation classifier (presently v11 and v12) does not incorporate control tissue of the posterior pituitary lobe as a distinct methylation class [6]. A match with the methylation class “pituicytoma/granular cell tumor/spindle cell oncocytoma” must therefore be interpreted as a match of unknown significance if neoplastic nature is in dispute. However, marked copy number alterations are deemed incompatible with non-neoplastic tissues and corroborate tumor diagnosis.

The case displayed numerous copy number changes, which also incorporated a loss of chromosome 1p and a gain of 1q. Of note, similar chromosomal aberrations and abundant copy number alterations have been described in 19% of pituicyte-derived tumors with SCO morphology and may be associated with shorter progression-free survival [4]. In challenging diagnostic situations, pituicyte-derived tumors without copy number alterations, accounting for 57% of cases, may yet be confirmed via detection of previously described mutations, for example affecting MAPK/PI3K pathway genes [4]. Unfortunately, DNA quantities were not sufficient to engage in further molecular analyses in the presented case.

In summary, we describe unusually widespread hemorrhage and necrosis in a pituicyte-derived tumor. Unlike in other common cystic lesions of the sellar region, these changes are exceptional in the pituicyte tumor family. Moreover, this report exemplifies benefits and limitations of epigenomic analyses in molecular diagnostics of posterior pituitary neoplasms. Careful interpretation of molecular findings is imperative when the neoplastic nature of a lesion is in dispute.

## Data Availability

The methylation data is available from the corresponding author upon request.
